# The Role of Probiotic Bacillus Spores and Amino Acids with Immunoglobulins on a Rat Enteropathy Model

**DOI:** 10.3390/biomedicines10102508

**Published:** 2022-10-07

**Authors:** Maria-Adriana Neag, Carmen-Stanca Melincovici, Adrian Catinean, Dana-Maria Muntean, Raluca-Maria Pop, Ioana-Corina Bocsan, Andrei-Otto Mitre, Mihai-Bogdan Cardos, Andreea-Ioana Inceu, Anca-Dana Buzoianu

**Affiliations:** 1 Department of Pharmacology, Toxicology and Clinical Pharmacology, Iuliu Hatieganu University of Medicine and Pharmacy, 400337 Cluj-Napoca, Romania; 2 Histology Discipline, Morphological Sciences Department, Iuliu Hatieganu University of Medicine and Pharmacy, 400349 Cluj-Napoca, Romania; 3 Department of Internal Medicine, Iuliu Hatieganu University of Medicine and Pharmacy, 400006 Cluj-Napoca, Romania; 4 Department of Pharmaceutical Technology and Biopharmaceutics, Iuliu Hatieganu University of Medicine and Pharmacy, 400012 Cluj-Napoca, Romania; 5 Faculty of Medicine, Iuliu Hatieganu University of Medicine and Pharmacy, 400349 Cluj-Napoca, Romania; 6 Emergency Clinical County Hospital Cluj-Napoca, 400006 Cluj-Napoca, Romania

**Keywords:** NSAIDs, probiotics, enteropathy, inflammation, microbiota

## Abstract

Non-steroidal anti-inflammatory drugs (NSAIDs) are some of the most widely used drugs due to their anti-inflammatory, analgesic and antipyretic pharmacological effects. Gastrointestinal side effects are some of the most severe and frequent side effects of NSAIDs. These depend on the balance of the gut microbiome, the abundance of Gram-negative bacteria, and the amount of lipopolysaccharide released. Therefore, restoring or improving gut bacteria balance with probiotic supplements could prove to be an adjuvant therapy against mild NSAID-induced enteropathy. Twenty-five Wistar albino male rats were divided into five groups. The negative control group was administered carboxymethylcellulose and the positive control group diclofenac (DIC), 8 mg/kg for 7 days, which represented the enteropathy model. Treatment groups consisted of a combination of pro-biotic spores (MSB), amino acids and immunoglobulins supplement (MM), which were also administered for 7 days. We analyzed hepatic injury markers (AST, ALT) and creatinine, and inflammatory markers, IL-6, TNF-α, PGE2, iNOS, as well as total antioxidant capacity. The results obtained in the present study suggest that the modulation of the intestinal microbiota by administration of probiotics (Bacillus spores), alone or in combination with immunoglobulins and amino acids, represents an attractive therapy for the prevention of NSAID-induced enteropathy.

## 1. Introduction

Nowadays, non-steroidal anti-inflammatory drugs (NSAIDs) are some of the most widely used drugs. Their widespread use is due to their anti-inflammatory, analgesic and antipyretic pharmacological effects. These effects are beneficial in the treatment of many diseases from rheumatic conditions to cardiovascular diseases. The most used NSAID is acetylsalicylic acid, not for the effects mentioned above but for the antiplatelet effect in patients with cardiovascular problems [1,2].

NSAIDs have various side effects, but for many drugs in this class (aspirin, indomethacin, naproxen, diclofenac), the most severe and frequent are the gastrointestinal side effects [3].

The possible mechanisms involved in the occurrence of these effects are: reduction in prostaglandin synthesis and destruction of the mucosal barrier function, binding of LPS and HMGB1 to TLR4 on macrophages with subsequent activation of pro-inflammatory pathways and release of cytokines such as TNF-α and IL-1B. The result is neutrophil infiltration of the mucosa and submucosa of the small intestine, and damage at this level [4].

Thus, the occurrence and severity of NSAID-induced intestinal damage depend on the abundance of Gram-negative bacteria and the amount of LPS released. Based on this information, it is hypothesized that restoring the balance of gut bacteria (correcting dysbiosis) may treat or prevent NSAID-induced damage [5,6].

The first data on the link between gut microbiota and NSAID enteropathy were reported by Robert et al. in 1977. This team observed that germ-free rats were resistant to indomethacin-induced damage in the small intestine. After that, other studies supported this hypothesis [7].

The beneficial effect of probiotics in intestinal diseases has been demonstrated in several studies: Bacillus spores, *Bifidobacterium longum BB536* in experimental colitis in rats [8,9], Bacillus spores in inflammatory bowel syndrome in patients [10], and *Bifidobacterium longum CECT7347* in gliadin-induced enteropathy in rats [11].

Probiotics are live microorganisms that help maintain and restore the microbial balance in case of dysbiosis. In this class of supplements, spore-based probiotics appear to be more effective than non-spore-based probiotics. This advantage is due to their resistance to harsh conditions such as stomach acids. In addition, they are stable at room temperature.

Regarding the efficacy of the spore-based probiotic formulation, it has been shown to reduce intestinal permeability, leading to a decrease in inflammatory cytokines. Thus, spore-based probiotics have a modulatory function in the microbiome [8]. The beneficial effects of Bacillus supplements may be the consequence of both actions: changing the composition of the intestinal microbiota and the production of SCFA (acetate, propionate, and butyrate), their main metabolites. SCFAs decrease intestinal inflammation and improve the integrity of the intestinal epithelial barrier [12,13].

Another nutraceutical agent with demonstrated gut health benefits is serum bovine immunoglobulin (SBI)-containing protein. The administration of SBI preparations has been shown to decrease the severity of enteropathy in animals because it improves intestinal barrier function and permeability [14]. Along with SBI, amino acids (threonine, serine, proline and cysteine) are important agents in maintaining the integrity of the intestinal mucosa and the balance of microorganisms in the gut. They can increase mucin production in the colon as well. This results in a thicker and healthier mucosal barrier [15,16].

Taking these into account, we considered that probiotic supplements (Bacillus spores) and amino acids with immunoglobulins would be a viable option for mild NSAID-induced enteropathy in experimental animals.

In our study, we evaluated the ability of a spore-based probiotic and a product containing amino acids with immunoglobulins (Microbiome Labs, Saint Augustine, FL, USA) to prevent intestinal damage associated with diclofenac-induced enteropathy in rats.

## 2. Materials and Methods

### 2.1. Ethical Considerations

The experimental study “The role of Bacillus spores in diclofenac-induced entheropathy in rats” was conducted according to “Guiding Principles in the Use of Animals in Toxicology” adopted by the Society of Toxicology (Reston, VA, USA) and all national laws regarding the protection of animals used for scientific research. The working animal protocol was revised and approved by the Ethics Committee of Iuliu Hatieganu University of Medicine and Pharmacy and by the National Sanitary Veterinary and Food Safety Authority (no. 247/18.02.2021).

### 2.2. Agents and Chemicals

We used MegaSporeBiotic^TM^ (MSB) probiotic capsules (Microbiome Labs, Saint Augustine, FL, USA), MegaMucosa^TM^ (MM) powder (Microbiome Labs, Saint Augustine, FL, USA), and Diclofenac (DIC) (solution 75 mg/3 mL). All products were administered orally as a suspension in 1 mL of 1% carboxymethylcellulose (CMC, vehicle). MSB contains spores from five Bacillus species (*B. licheniformis*, *B. indicus*, *B. subtilis*, *B. clausii*, and *B. coagulans*) and MM is a combination of amino acids (L-proline, L-serine, L-cysteine, L-threonine), serum-derived Ig concentrate, and bioflavonoids.

### 2.3. Animals

Twenty-five (*n* = 25) Charles River Wistar albino male rats weighing between 230 and 280 g were obtained from the Center for Experimental Medicine and Practical Skills of Iuliu Hatieganu University of Medicine and Pharmacy. The rats were maintained under standard conditions of light (12 h light/dark cycles), temperature (22 ± 2 °C), and humidity. The animals were fed rat chow ad libitum and had free access to tap water. The rats were acclimated under these conditions for three days prior to starting the experiment.

The rats were divided into 5 groups, with 5 rats per group.

### 2.4. Experimental Design

Group I received the vehicle (1% CMC), serving as the control group;

Group II received 1% CMC and DIC (8 mg/kg body weight), serving as the enteropathy model;

Group III received MSB (1 × 10^9^ colony forming units (CFU)/day and DIC (8 mg/kg body weight);

Group IV received MM (700 mg/kg/day) and DIC (8 mg/kg body weight);

Group V received MSB (1 × 10^9^ CFU/day), MM (700 mg/kg/day) and DIC (8mg/kg body weight).

All treatments were administered orally through a feeding tube for 7 days by gavage. MSB and MM were administered one hour before diclofenac administration.

On day 8 (at 24 h after the last administration of treatments), blood, small intestine and liver samples were collected for further analysis. Blood was collected from the retro-orbital (periorbital) sinus plexus under anesthesia. Serum was separated by centrifugation (4000 rpm, 15 min, room temperature) and then stored at −20 °C for further biochemical analysis.

### 2.5. Evaluation of Inflammatory Markers, Biochemical Parameters and Total Antioxidant Capacity

Aspartate aminotransferase (AST), alanine aminotransferase (ALT) and creatinine were measured by automated biochemical analysis according to the BioSystems A15 instrument protocol. Serum levels of the pro-inflammatory cytokines IL-6, TNF-α, PGE2, iNOS were quantified by the ELISA technique (enzyme-linked immunosorbent assay) using commercially available ELISA kits (Rat TNF-α and IL 6 Standard ABTS ELISA Development Kits; PeproTech Inc., Rocky Hill, NJ, USA and Rat PGE2 and NOS2/iNOS from Elabscience, Houston, TX, USA). AST and ALT were expressed in U/L and creatinine in mg/dl. TNF-α, IL-6, and PGE2 values were expressed in pg/mg liver tissue, whereas NOS2/iNOS values were expressed in ng/mg liver tissue.

Total antioxidant capacity (TAC) levels were measured according to a validated method previously described by Erel [17]. The test is based on the ability of antioxidants to decolorize the blue-green reagent, the radical cation 2,2′-azinobis-(3-ethylbenzothiazoline-6-sulfonic acid) (ABTS+). The color depends on the concentration of antioxidants and their antioxidant capacities. The reduced ABTS molecule is oxidized to ABTS+ using only hydrogen peroxide in an acidic medium (acetate buffer: 30 mmol/L, pH 3.6). In acetate buffer, concentrated ABTS+ molecules (deep green) remain stable for a long time. Slow color bleaching depends on dilution with a more concentrated acetate buffer solution at a high pH (high pH acetate buffer: 0.4 mol/L, pH 5.8). The bleaching rate is proportional to the antioxidant concentration and inversely related to the TAC of the sample. This reaction can be monitored spectrophotometrically at 660 nm. Trolox was used to generate the calibration curve. This is widely used as the standard for TAC assays, and results are expressed in Trolox mmol/L equivalents.

### 2.6. Histological Assessment

For histopathological examination, harvested intestine segments were preserved in 10% formaldehyde, dehydrated in ethanol, and embedded in paraffin. Sections of 7 µm thickness were prepared, and then they were deparaffinized, rehydrated with ethanol solutions in decreasing concentrations (100%, 90%, 80%), and stained with hematoxylin-eosin. Histological evaluation was performed with a Leica DM750 Clinical microscope (Leica Microsystems, Germany), and images were obtained with a Leica ICC 50 W microscope camera (Leica Microsystems, Germany).

### 2.7. Statistical Analyses

To describe the data, we used bar graphs representing the averages of the variables per group and the standard deviations. Since our data are of continuous quantitative type, in the data analysis process we used the Shapiro–Wilk data normality test, where a *p* > 0.05 is representative of normally distributed data. The next step consisted of applying the test to assess differences in the ANOVA data. Results with statistically significant ANOVA test were further analyzed by Student’s *t*-tests with Tukey’s correction for multiple testing. The results were considered statistically significant for a *p* < 0.05. Data analysis was performed using GraphPad Prism, version 8.0.0 for Windows, GraphPad Software, San Diego, CA, USA, www.graphpad.com.

## 3. Results

All analyzed variables (PGE2, iNOS, IL-6, TNF-α and creatinine) were normally distributed (Shapiro–Wilk *p* > 0.05) for each analyzed group.

Descriptive results from each variable of the groups included in the study are shown below.

**Figure 1:**
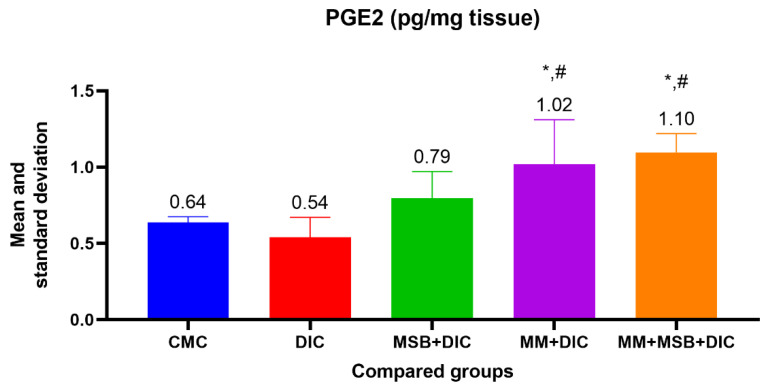
The mean value and standard deviation of PGE2 between the analyzed groups. Group I-CMC, negative control group; group II-DIC, disease control group; group III-MSB+DIC, group receiving MegaSporeBiotic; group IV-MM+DIC, group receiving MegaMucosa; group V-MM+MSB+DIC, group receiving MegaSporeBiotic and MegaMucosa; *, *p* < 0.05 compared with group II (DIC); #, *p* < 0.05 compared with group I (CMC).

For PGE2, the ANOVA test was statistically significant (*p* < 0.001), and between-group analysis revealed a statistically significant difference between group I and group IV (*p* = 0.02, mean difference = 0.38), group I and group V (*p* = 0.003, mean difference = 0.46), group II and group IV (*p* = 0.002, mean difference = 0.48) and between group II and group V (*p* < 0.001, mean difference = 0.56) (Figure 1).

**Figure 2:**
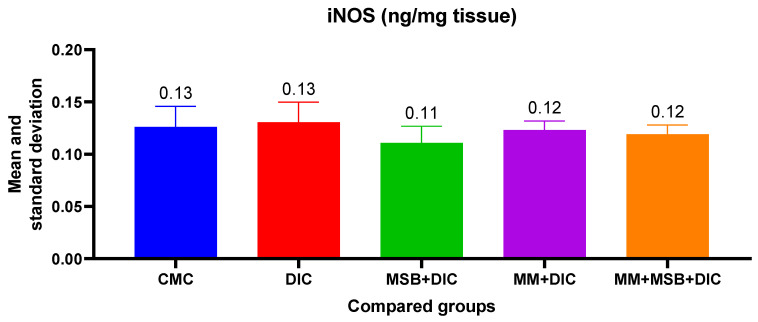
The mean value and standard deviation of iNOS between the analyzed groups. Group I-CMC, negative control group; group II-DIC, disease control group; group III-MSB+DIC, group receiving MegaSporeBiotic; group IV-MM+DIC, group receiving MegaMucosa; group V-MM+MSB+DIC, group receiving MegaSporeBiotic and MegaMucosa.

For the iNOS variable, the ANOVA test was statistically insignificant (*p* = 0.34); thus, there is no statistically significant difference between the groups (Figure 2).

**Figure 3:**
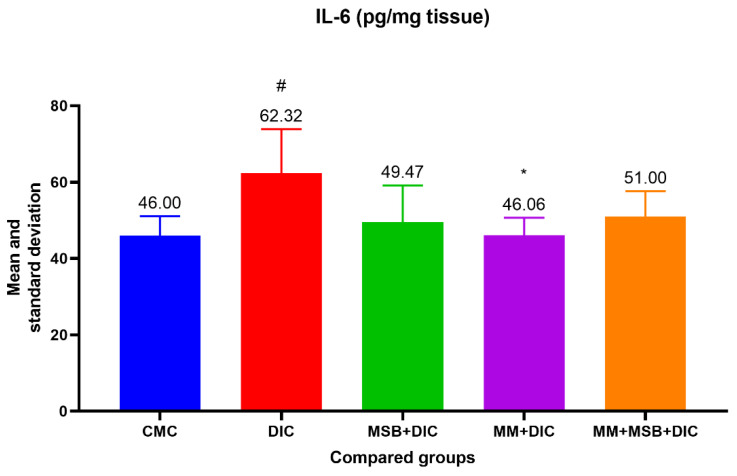
Mean value and standard deviation of IL-6 between analyzed groups. Group I-CMC, negative control group; group II-DIC, disease control group; group III-MSB+DIC, group receiving MegaSporeBiotic; group IV-MM+DIC, group receiving MegaMucosa; group V-MM+MSB+DIC, group receiving MegaSporeBiotic and MegaMucosa; *, *p* < 0.05 compared with group II (DIC); #, *p* < 0.05 compared with group I (CMC).

The ANOVA test of variances for the IL-6 variable was statistically significant (*p* = 0.02). In the test for evaluating the differences between groups, we detected a statistically significant difference between group I and group II (*p* = 0.03, difference in means = 16.32) and between group II and group IV (*p* = 0.03, difference in means = 16.26) (Figure 3).

**Figure 4:**
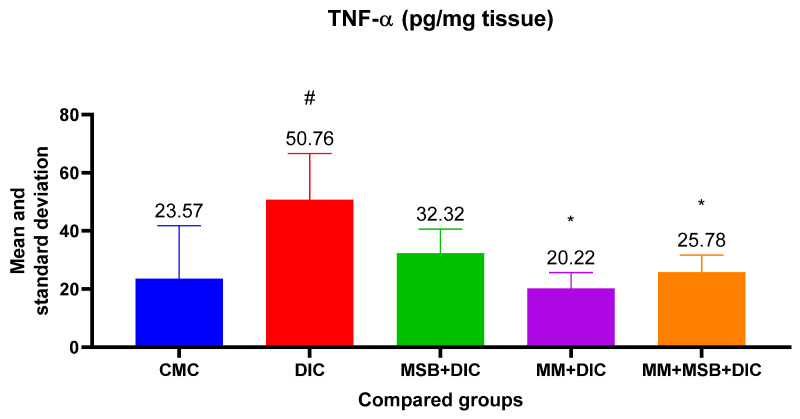
Mean value and standard deviation of TNF-α between analyzed groups. Group I-CMC, negative control group; group II-DIC, disease control group; group III-MSB+DIC, group receiving MegaSporeBiotic; group IV-MM+DIC, group receiving MegaMucosa; group V-MM+MSB+DIC, group receiving MegaSporeBiotic and MegaMucosa; *, *p* < 0.05 compared with group II (DIC); #, *p* < 0.05 compared with group I (CMC).

For TNF-α, the ANOVA test was statistically significant (*p* = 0.005). Analysis between groups revealed statistically significant differences between the means of group I and group II (*p* = 0.01, mean difference = 27.19), group II and group IV (*p* = 0.005, mean difference = 30.55) and group II and group V (*p* = 0.03, mean difference = 24.98) (Figure 4).

**Figure 5:**
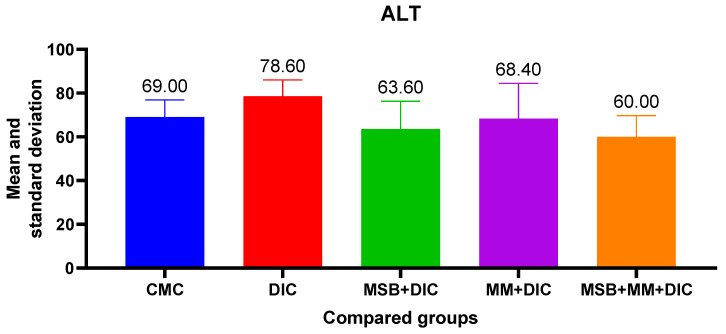
Mean value and standard deviation of ALT between analyzed groups. Group I-CMC, negative control group; group II-DIC, disease control group; group III-MSB+DIC, group receiving MegaSporeBiotic; group IV-MM+DIC, group receiving MegaMucosa; group V-MM+MSB+DIC, group receiving MegaSporeBiotic and MegaMucosa.

**Figure 6:**
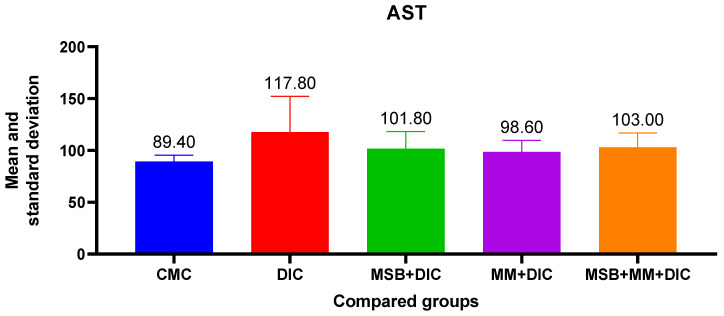
Mean value and standard deviation of AST between analyzed groups. Group I-CMC, negative control group; group II-DIC, disease control group; group III-MSB+DIC, group receiving MegaSporeBiotic; group IV-MM+DIC, group receiving MegaMucosa; group V-MM+MSB+DIC, group receiving MegaSporeBiotic and MegaMucosa.

Administration of diclofenac has a negative impact on liver function, evidenced by the increase in AST, ALT and creatinine. These increases, although statistically insignificant (in the ANOVA test *p* > 0.05), were more evident in the case of ALT (31.77%) vs. AST (10.96%). The probiotic supplement administered in combination with MegaMucosa had a beneficial effect, especially on ALT where it reduced the increase by 23.66% (Figure 5 and Figure 6).

For the creatinine data, the ANOVA test was statistically insignificant (*p* = 0.09); therefore, there were no statistically significant differences between the studied groups.

**Figure 7:**
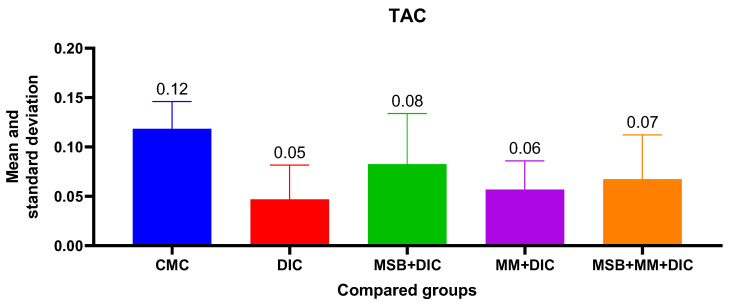
Mean value and standard deviation of TAC between analyzed groups. Group I-CMC, negative control group; group II-DIC, disease control group; group III-MSB+DIC, group receiving MegaSporeBiotic; group IV-MM+DIC, group receiving MegaMucosa; group V-MM+MSB+DIC, group receiving MegaSporeBiotic and MegaMucosa.

For TAC, the ANOVA analysis was statistically insignificant (*p* = 0.07), with no statistically significant differences between groups. However, the administered substances, either alone or in combination, reduce the decrease in TAC from 58.33% in the case of group II to 50% for group IV, 41.67% for group V, and 33.33% for group III (Figure 7).

**Figure 8:**
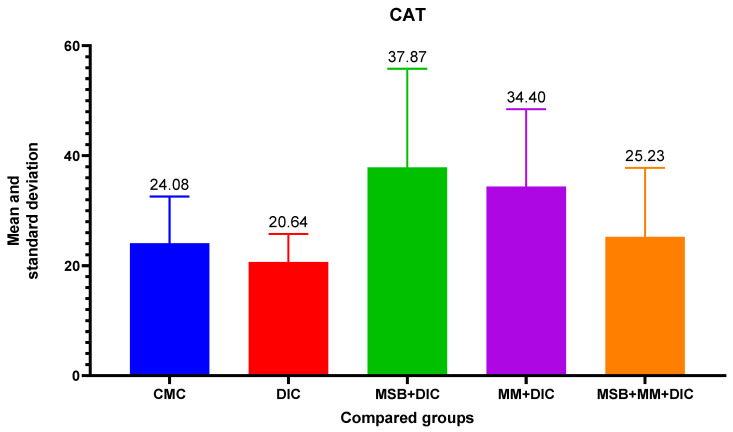
Mean value and standard deviation of CAT between analyzed groups. Group I-CMC, negative control group; group II-DIC, disease control group; group III-MSB+DIC, group receiving MegaSporeBiotic; group IV-MM+DIC, group receiving MegaMucosa; group V-MM+MSB+DIC, group receiving MegaSporeBiotic and MegaMucosa.

For differences between groups for CAT, ANOVA analysis did not reveal a difference between group variances (*p* = 0.18). Therefore, there is no statistically significant difference in the distribution of the CAT variable between the groups. However, CAT increases upon administration of MSB, MM, or the combination to significantly higher values than in the negative control group (Figure 8).

### Histologic Examination

**Figure 9:**
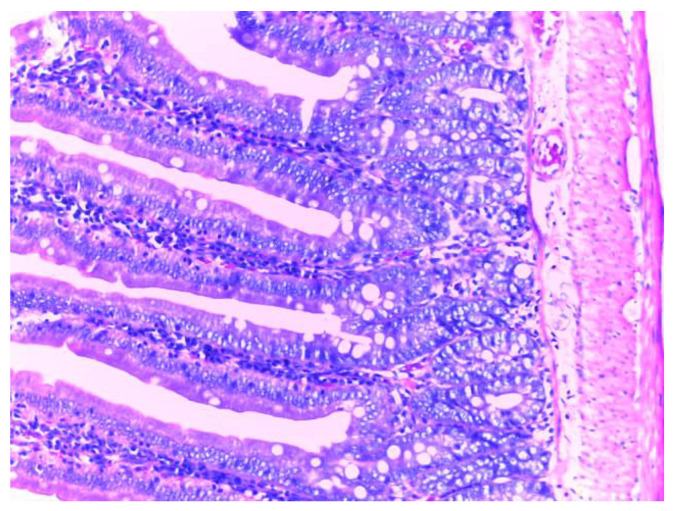
Normal histological appearance of the small intestine in group I (H&E stain, ob 20X).

The control group (group I) shows a normal structure of the small intestine with intestinal villi, numerous simple tubular intestinal glands lined by polymorphic glandular epithelium, and lamina propria (chorion) with loose connective tissue and a discrete inflammatory infiltrate. The muscularis mucosae, submucosa, tunica muscularis and serosa have a normal histological appearance (Figure 9).

**Figure 10:**
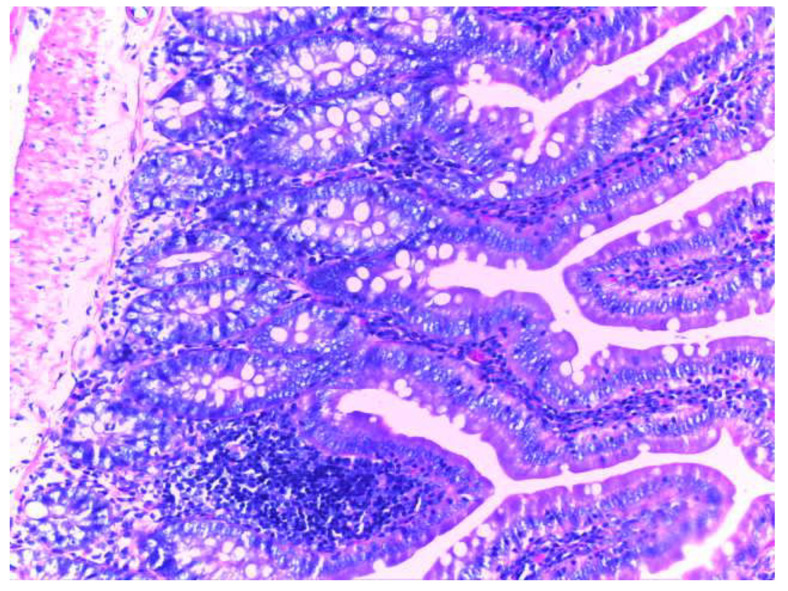
Histological appearance of the small intestine in animals in group II (H&E stain, 20X).

Histological examination of the small intestine in animals treated with diclofenac (group II) revealed an abundant inflammatory infiltrate, even with small lymphoid follicles and few neutrophils in the lamina propria. No mucosal erosions are observed, and the submucosa, tunica muscularis and serosa have a normal histological appearance (Figure 10).

**Figure 11:**
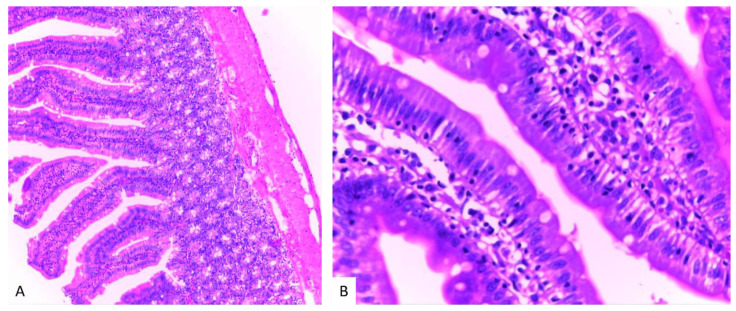
Histological examination of group III (H&E stain, 10X (**A**); 40X (**B**)).

**Figure 12:**
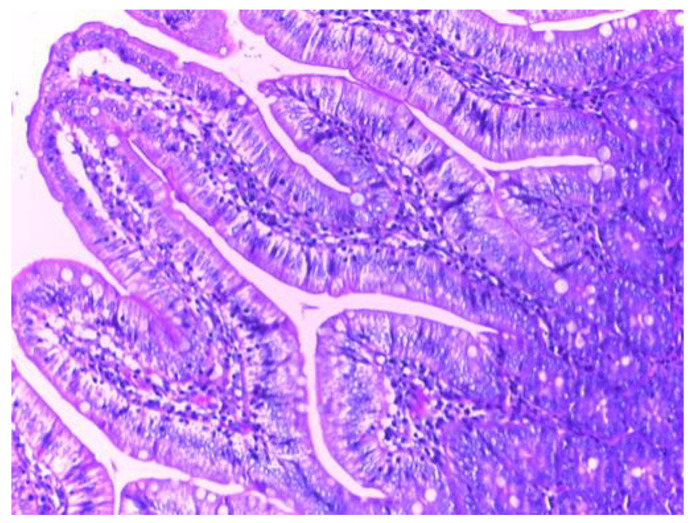
Histological examination of group IV (H&E stain, 20X).

**Figure 13:**
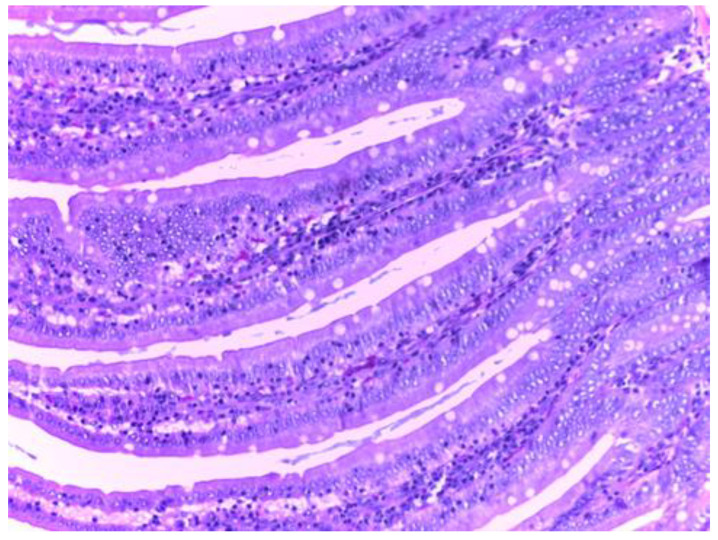
Histological examination of group V (H&E stain, 20X).

Histological analysis in groups treated with MSB, MM or their combination (group III, IV, V), revealed no visible injuries to the structure of intestinal mucosa or other layers of the intestinal wall. (Figure 11, Figure 12 and Figure 13).

## 4. Discussion

Through the present study, we aimed to provide new evidence regarding the protective effect of probiotics containing a combination of Bacillus spores either alone or in combination with an immunoglobulin and amino acid supplement in mild NSAID-induced toxicity, especially in the intestinal environment. Diclofenac has been shown to cause intestinal damage and increase intestinal permeability and bacterial translocation. This NSAID also alters the composition of the intestinal microbiota, increasing the abundance of Proteobacteria and Bacteroides and decreasing Firmicutes in the rat ileum [18,19].

It is known that PGs have an important role in maintaining blood flow at the gastrointestinal level and also an essential role in mucus production. Administration of NSAIDs causes a reduction in PG levels and damage to the small intestine [20].

In our study, a statistically significant decrease in PG level is observed in the group treated only with diclofenac, in contrast with a significant increase in the groups treated with a combination of diclofenac and MSB, MM or MSB and MM. The role of intestinal bacteria in the induction of enteropathy is recognized in many studies. Some experimental studies have showed that germ-free animals (rodents) have a minimal or even absent risk of developing intestinal injury when taking NSAIDs, but after bacterial colonization with Gram-negative bacteria, the susceptibility of the small intestine to post-NSAID lesions increases significantly [21].

iNOS expression is known to be modulated by cytokines. They play an important role in regulating the immune system, a system that contains both pro-inflammatory and anti-inflammatory cytokine factors. Probiotics have been shown to inhibit the activation of Nf-kB, an activation that triggers inflammatory mechanisms with the increase in adhesion molecules and pro-inflammatory cytokines. The activation of iNOS and COX-2 is also influenced by this pathway [22].

In the present study, diclofenac did not cause significant raised expression of iNOS in liver tissue, and MSB and MM prevented the expression amplification, but the values did not have statistical significance. Changes in the iNOS levels also depend on the tissue in which they are determined. It is possible that the changes are further expressed at the level of the renal tissue, and particularly at the level of the renal cortex. However, not all NSAIDs cause significant changes in iNOS expression. For example, aspirin and celecoxib do not cause significant changes in iNOS at the myocardial level [23].

TNF-α and IL-6 are pro-inflammatory cytokines that can disrupt the immune system and amplify inflammation. The results of our study show that the TNF-α level increased significantly in the diclofenac group, and the studied substances (MSB, MM) or their combination prevented the increase, which translates into a protective effect of these substances.

Differences between groups regarding IL-6 levels showed statistical significance only between the group with diclofenac and the one treated with MM. However, we observed a 20.62% decrease in the mean of IL-6 in the group treated with MSB compared with the group that received only diclofenac. Looking at the values obtained, both for TNF-α and for IL-6, the anti-inflammatory effect of MM can be deduced, as well as the moderate improvement of inflammation in the groups treated with MSB or MSB+MM. Although some of these results are consistent with the changes observed in the histopathological analysis, similar studies conducted in larger groups of rats could confirm these hypotheses which in the present case are devoid of statistical significance.

During cellular respiration, reactive oxygen species are generated in the body. Under physiological conditions, the body’s antioxidant systems have the ability to rapidly neutralize these compounds. In pathological conditions, however, antioxidant systems are overwhelmed, and there is a tendency to accumulate reactive species, with destructive consequences on proteins [8].

Oxidative stress is a condition frequently associated with intestinal damage, especially in the presence of NSAID-induced enteropathy [24].

No statistically significant results were obtained when we measured the parameters for oxidative stress (total antioxidant capacity). However, the administered substances, either alone or in combination, reduce the decrease in TAC from 58.33% in the case of diclofenac administration, to 50% for the group treated with DIC and MM, 41.67% for the group treated with DIC, MM and MSB, and even 33.33% for DIC and MSB. These results are encouraging for future research on larger groups of animals.

In addition to intestinal toxicity, diclofenac also causes liver and kidney toxicity. This has been evidenced by increased liver enzymes (AST, ALT) and increased creatinine. Hepatotoxicity has been observed and objectified by increased liver enzymes in other studies as well, showing the presence of injury at the hepatocyte level [21].

Administration of MSB, MM, or the combination resulted in reduced increases in AST and ALT. This beneficial effect is not statistically significant, but we must take into account the limitations of the study. For ALT, the values decreased below those of the control group after administration of the test substances. The hepatoprotective effect of Bacillus spores was also demonstrated in another experimental study that investigated the effect of this supplement in paracetamol-induced hepatotoxicity [25].

The histopathological results support the obtained biochemical results. The analysis of the intestine tissue sections showed that in the animals treated only with diclofenac, there is an abundant inflammatory infiltrate with the presence of neutrophils in the chorion structure of the intestinal mucosa. However, no mucosal erosions were observed, and the underlying structures retained their normal appearance. This may be due to either the inadequate duration of NSAID exposure or the low dose used, although these were consistent with those used in the literature.

NSAID therapy, especially in some chronic inflammatory diseases, is recommended for long-term use. The duration of therapy is directly proportional to the risk of adverse reactions. For this reason, it is necessary to prevent the injuries induced by this pharmacotherapeutic class in the long term with safe and effective substances [26].

Additionally, the cardiovascular risk of COX-2 selective NSAIDs limits their use and therefore the recommendations for classic NSAIDs remain high [27].

## 5. Conclusions

The results obtained in the present study suggest that the modulation of the intestinal microbiota by administration of probiotics (Bacillus spores), alone or in combination with immunoglobulins and amino acids, could represent an attractive therapy for the prevention of NSAID-induced enteropathy. Further studies, both experimental and in human subjects, are needed to support the results obtained in the current study.

## Data Availability

Not applicable.
